# Clinical features and viral load variations of Mpox: a retrospective study in Chongqing, China

**DOI:** 10.1186/s12879-024-09537-0

**Published:** 2024-06-26

**Authors:** Sen Yang, Chao Xia, Yuxin Zhang, Yan Shen, Chengshuang Xia, Yanqiu Lu, Shifang Su, Changgang Deng, Vijay Harypursat, Jing Wang, Jing Yuan, Yemiao Chen

**Affiliations:** 1 Biobank, Chongqing Public Health Medical Center, Chongqing, 400036 China; 2 Department of Infectious Diseases, Chongqing Public Health Medical Center, Chongqing, 400036 China; 3 Department of Disease Prevention, Chongqing Public Health Medical Center, Chongqing, 400036 China; 4 Department of Medical Laboratory, Chongqing Public Health Medical Center, Chongqing, 400036 China

**Keywords:** Mpox, HIV, Infection, Clinical features, Viral load

## Abstract

**Purpose:**

Since May 2022, Mpox has spread extensively outside of Africa, posing a serious threat to the health of people globally, and particularly to the men who have sex with men (MSM) population. Chongqing, a province in Southwest China, has relatively large MSM and people living with HIV (PLWH) populations, presenting conditions conducive to the wide dissemination of Mpox. In this study, we investigated the clinical characteristics of Mpox patients among MSM and PLWH in Chongqing, aiming to inform the development of targeted prevention, control, and treatment strategies for Mpox.

**Method:**

We evaluated the clinical characteristics, travel history, time of onset, distribution and number of skin lesions of Mpox patients admitted to the Chongqing Public Health Medical Center between September 2022 and October 2023. Meanwhile, a series of clinical samples were collected and the pathogen of interest was identified as Mpox virus using quantitative polymerase chain reaction (qPCR). The results were presented in the form of cycle thresholds (Ct), which help to approximate the quantification of viral load.

**Results:**

As of October 11, 2023, the Chongqing Public Health Medical Center reported a total of nine Mpox virus infections. All the patients identified were male and belonged to the MSM population, among whom seven (77.8%) were living with HIV, and maintained a preserved immune system while achieving viral suppression via effective ART. We observed no discernible clinical differences between MSM with Mpox with or without HIV, and no fatalities were recorded. Viral loads were observed to be higher in samples taken from the skin than those from the throat, nasopharynx, blood, or semen.

**Conclusion:**

In this retrospective study, the clinical manifestations of MPXV infection appeared consistent among MSM patients, regardless of HIV status. Elevated MPXV viral loads in the skin and mucosal tissues, particularly at genital and anal sites, indicate that transmission is more likely to occur via direct physical contact as opposed to respiratory pathways or through exposure to bodily fluids.

## Introduction

The Mpox virus (MPXV) is a zoonotic, double-stranded DNA virus that causes Mpox, a disease that is similar to, but produces a milder disease than, smallpox [1]. MPXV was first described in 1958, and the first case was reported in 1970 in a 9-month-old boy in the Democratic Republic of the Congo [2, 3]. Symptoms of Mpox are characterized by a triad of fever, lymphadenopathy, and rash. Complications such as encephalitis or extensive lesions of the skin and mucous membranes are rare [4]. Consequently, mortality rates for Mpox are relatively low, ranging from 0 to 10% for cases reported after 2000 [5]. Prior to the global outbreak of Mpox in 2022, Mpox was primarily confined to ten countries within Africa [6]. On the 7th of May 2022, the United Kingdom reported a confirmed case of Mpox in a person who had returned from a trip to Nigeria. This was followed by reports of Mpox cases spreading across Europe, the Americas, Africa, and Asia [7]. According to the US Centers for Disease Control and Prevention, as of November 8, 2023, the total number of confirmed Mpox cases reported globally was 91,417, with a total of 166 deaths [8]. This marks the largest and most widespread Mpox outbreak outside of Africa to date.

MPXV has contemporarily been classified into the West African and the Central African clades, reflecting variations in MPXV virology, epidemiology, and clinical aspects of diagnosis [9]. Typically, the incubation period for MPXV spans from six to 13 days, though this can extend up to 21 days in some cases [4, 10]. Initial infection with Mpox is marked by non-specific symptoms, including back pain, headache, chills, fever, fatigue, myalgia, lethargy, and lymphadenopathy, which last from 0 to 5 days [11]. Approximately 95% of patients experience a resolution of fever after three days, coinciding with the emergence of a papular rash that spreads centrally, notably affecting the face in 95% of cases, and the palms and soles in 75% of cases [12]. MPXV may be identified at various body sites, including the skin, throat, blood, semen, and the anal region [13]. During the 2022 outbreak, sexual transmission was highlighted as a principal vector for the multinational spread of MPXV, and particularly identified sexual intercourse as a significant risk factor [14]. This mode of transmission shares a similarity to that of HIV, contributing to a relatively higher incidence of Mpox among people living with HIV (PLWH), especially among men who have sex with men (MSM) [3]. The prognosis for individuals having concurrent MPXV and HIV infections remains a subject of debate. Some research suggests that patients co-infected with MPXV and HIV may experience a more protracted illness, develop more severe skin lesions, and have a higher likelihood of developing secondary bacterial infections [15, 16]. However, other studies indicate that the overall clinical presentation of MPXV does not materially differ between HIV-positive and HIV-negative MSM [17]. Thus, the dynamics of MPXV-HIV co-infection warrant further investigative scrutiny and ongoing global vigilance.

Due to the strict enforcement of COVID-19 border quarantine measures and policies mandating isolation for inbound travelers to mainland China, the first imported case of Mpox in China was documented relatively later than the rest of the world, which was in Chongqing, a province in Southwest China in September 2022 [18]. The affected patient was successfully treated and discharged from our hospital (Chongqing Public Health Medical Center) in October of the same year. Since June 2023, mainland China has witnessed a modest increase in Mpox cases, with the number of affected individuals exceeding 1,400. Among these, there was one case reported in September 2022, followed by a monthly breakdown from June to September 2023 of 106, 491, 501, and 306 cases, respectively. Of the total Mpox patients, 1,398 were male and 7 were female. Notably, over 95% of the cases involved MSM, encompassing all Mpox cases reported from September 2022 through September 2023 [19]. Despite this rapid increase in case numbers, the clinical manifestations and viral load fluctuations associated with Mpox infection in mainland China have not as yet been described, nor has there been research comparing these characteristics with those observed in outbreaks in other countries around the world. Concurrently, Chongqing is facing a serious HIV epidemic and is also home to a large MSM community; these are factors that could potentially facilitate the easy transmission and dissemination of Mpox within Chongqing and subsequently throughout China. Therefore, a deeper investigation into the disease attributes of Mpox within this demographic is imperative.

In our study, we conducted a retrospective analysis of Mpox cases in Chongqing, China, utilizing the stored samples and data from the Biobank of Chongqing Public Health Medical Center. Our aim was to outline the clinical characteristics and the viral loads of these cases, thereby laying a foundational framework for informing future strategies for Mpox epidemic prevention, control, and treatment efforts.

## Materials and methods

### Study design

This retrospective case study enrolled patients at the Chongqing Public Health Medical Center in China who were confirmed to have MPXV infections from September 14, 2022. Our study involved the identification of Mpox DNA in stored samples using quantitative real-time polymerase chain reaction (qPCR) technology. Outcomes are reported as cycle threshold (Ct) values, which enable an estimation of viral loads in individuals in our cohort.

### Data Collection and procedures

The 9 laboratory confirmed Mpox cases were collected from the Chongqing Public Health Medical Center between September 2022 and October 2023. The following data and background information were gathered: the date of MPXV infection diagnosis, the probable country of origin of infection (including any travel history), the onset of symptoms, the location and number of lesions, changes in viral load, history of smallpox vaccination, and clinical severity of the Mpox disease. The severity assessment included the categorization of the number of lesions, the progression of symptoms, and the likely general symptoms, such as fever, head and extremity aches, night sweats, and lymphadenopathy.

Additionally, samples had been collected including blood from 4 patients, oropharyngeal from 2 patients and nasopharyngeal swabs from 6 patients, urine from 1 patient, feces from 1 patient, anus swabs from 4 patients, semen from 1 patient, and environmental samples (walls, floors, doorknobs, stretchers, computers, etc.) from 4 patients. All specimens were handled in our biosafety level 2 virology laboratory. DNA extraction of clinical samples was performed using the EXM6000 magnetic bead method nucleic acid extraction instrument (ZYBIO Co. Ltd., China). Real-time MPXV PCR assays were performed on CFX96 platform (Bio-Rad, America). Fluorescence quantitative PCR was performed with the nucleic acid testing kit (DAAN Gene Co. Ltd. China) for MPXV detection. The PCR protocols were developed with minor adjustments to the initial protocols reported previously [20].

## Results

### Epidemiological characteristics

From September 14, 2022, to October 10, 2023, the Chongqing Public Health Medical Center reported a total of nine PCR-confirmed cases of MPXV infection (Table 1). The cohort under study comprised exclusively young adult men who have sex with men (MSM), having a median age of 30 years, with ages ranging from 20 to 41 years. Each individual was sexually active with another man within the month preceding their diagnosis. Patient 1 contracted the virus in Germany, while Patient 2 did so in Thailand. Patient 3 was infected through contact with Patient 2. The subsequent six patients (Patients 4 to 9) had not traveled outside of Chongqing; however, they confirmed to have had engaged in sexual activities with other men. Specifically, Patient 5 confirmed having sexual relations with a man 16 days before his hospital admission.

**Table 1:** Clinical and epidemiological characteristics of studied Mpox patients

	Pat. 01	Pat. 02	Pat. 03	Pat. 04	Pat. 05	Pat. 06	Pat. 07	Pat. 08	Pat. 09
Age (years)	29	32	38	20	24	41	25	30	32
HIV status	Neg	Pos	Pos	Pos	Neg	Pos	Pos	Pos	Pos
CD4 counts	789	560	1164	517	522	137	416	366	369
ART drugs	NA	DTG/3TC	FTC/TAF/BIC	FTC/TAF/BIC	NA	DTG/3TC	FTC/TAF/BIC	DTG/3TC/AZT	FTC/TAF/BIC
HIV-RNA	NA	ND	ND	ND	NA	ND	ND	ND	ND
Concomitant STIs	Neg	Neg	Neg	Neg	Neg	Neg	HCV	Neg	Neg
Epidemiological link (Travel/contact)	Spain and Germany/Yes	Thailand/Yes	No/Yes	No/Yes	No/Yes	No/No	No/No	No/No	No/No
Localization of skin lesions	Face, oral arms, legs, perianal area, genital area and trunk	Perianal area	Groin	Neck and buttock	Face, arms, legs trunk and perineum	Hands, Face, trunk and perianal area	Back	The whole body	Face, neck and perianal area
Number of lesions	34	3	1	4	NA	NA	2	NA	12
Smallpox/Mpox vaccination	No	No	No	No	No	No	No	No	No

NA: not available; ND: not detected; ART: antiretroviral therapy; FTC: emtricitabine; TAF: tenofovir alafenamide; BIC: bictegravir; DTG: dolutegravir; 3TC: lamivudine; AZT: zidovudine; HIV: human immunodeficiency virus; HCV: hepatitis C virus; Neg: negative; Pos: positive; Pat.: patient; STI: sexually transmitted infection. Epidemiological links were investigated. Travel: History of travel outside Chongqing in the last month/ contact: history of close physical contact with a MPXV positive case

### Clinical appearance

**Fig. 1:**
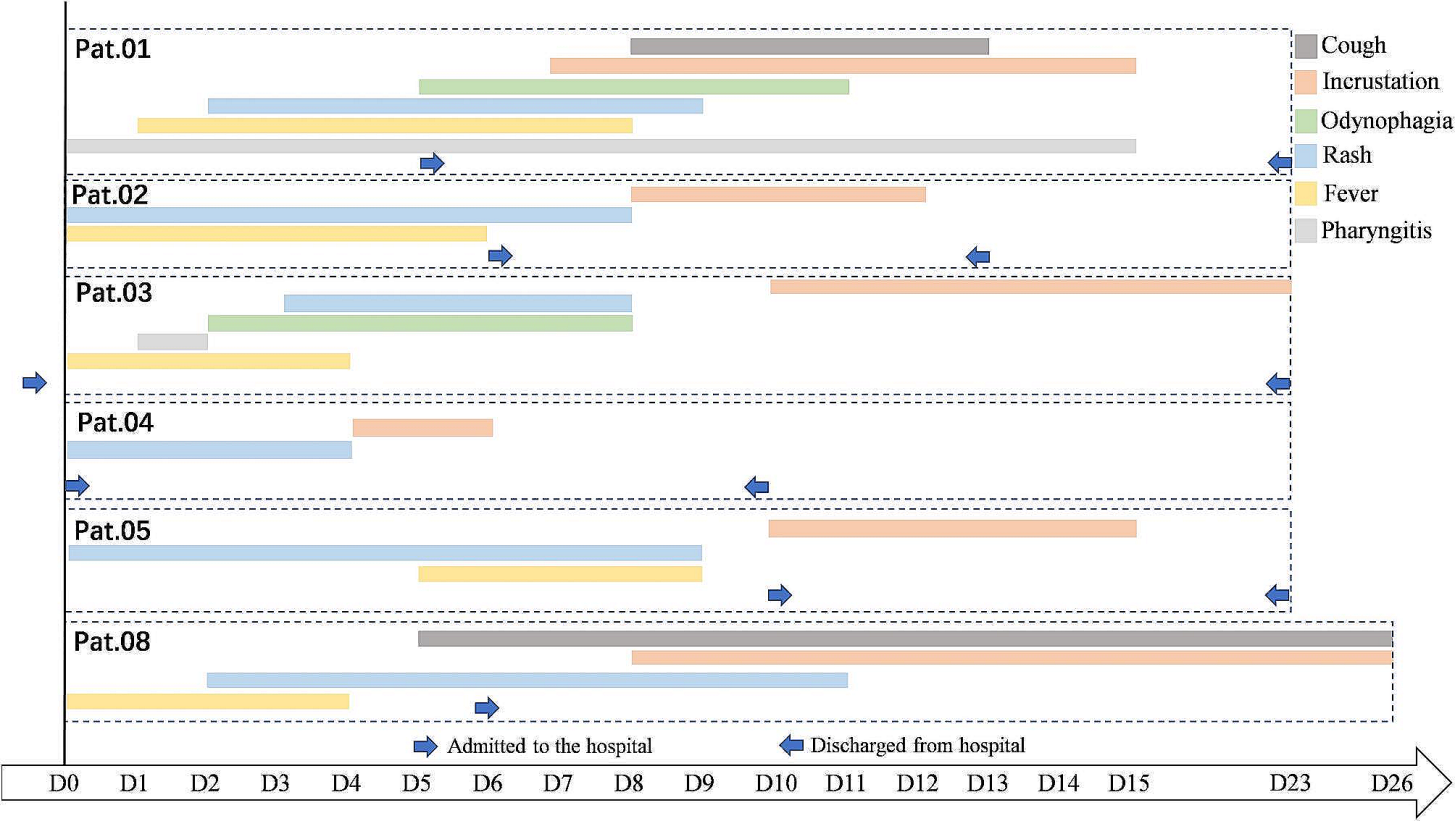
Time series of symptom onset and hospitalization time of six Mpox cases D0 = day 0, the day of onset of symptoms, D1 = day 1, one day from onset of symptoms, and etc. The time between the two blue arrows is the length of hospital stay

**Fig. 2:**
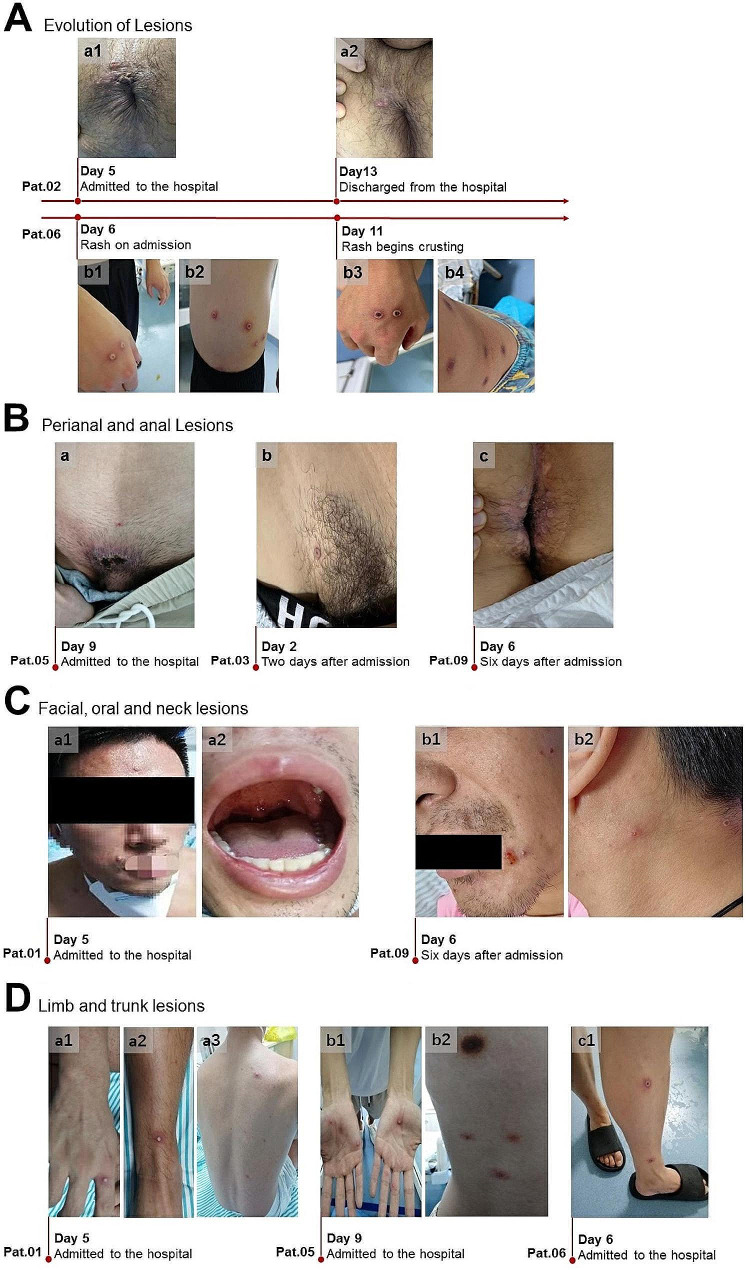
Skin presentation of lesions in Mpox patients (**A**) Disease progression in two patients with HIV. In Pat.02 (**A**-**a**), three pale red maculopapular eruptions were observed around the anus; one was pustular and one was ulcerated, and there was no scab. Eruptive blistery papules could be seen on the entire body of Pat.06 (**A**-**b**), and no obvious rupture was observed. (**B**–**D**) Photographs of lesions (lesions of the skin and mucous membranes) in multiple patients with and without (Pat.01, Pat.05) HIV

The majority of cases presented with relatively mild symptoms. There were no fatalities reported. As illustrated in Fig. 1, the median time from the onset of symptoms to the identification of Mpox virus (MPXV) was five days, with a range from immediate diagnosis to ten days. The median hospital stay for patients was 18 days, and ranged from seven to twenty-four days. The most frequently reported symptoms included fever, cough, headache, and either a dry throat or sore throat. Predominantly, Mpox lesions presented in the anal or genital regions, as depicted in Fig. 2A-B. None of the patients demonstrated necrotic evolution of pre-existing lesions after admission. Patient 2 was admitted on day five after symptom onset and was discharged from hospital on day 13, after healing of perianal lesions (Fig. 2A-a). On admission, Patient 6 had sparsely disseminated papular eruption and no visible ulcers. By day 11, the rashes on her hands and torso had crusted (Fig. 2A-b). Only three patients developed lesions on the face, trunk, and limbs (Fig. 2C-D). There were no discernible differences in the clinical appearance of the Mpox rash between patients positive or negative for HIV (Fig. 2B-a, C-a and D-a, b). For the majority of our cohort, the infection was localized, and included one presentation with more than a single lesion. There was one case with a more severe presentation, characterized by the presence of 34 lesions (Table 1). As China ceased routine smallpox vaccination in 1981 [21] and considering the limitations of current Mpox vaccines, none of the patients had received vaccination against Mpox (Table 1).

### Immune characteristics and medication of patients

The majority of patients were young and in generally good health. Patients 1 and 5 were not HIV positive, while the others in our cohort were living with HIV (Table 1). Notably, Patient 1 exhibited more severe symptoms, whereas the symptoms were relatively mild in the remaining eight patients. Among the seven HIV-positive patients, six had normal immune functioning, as indicated by a median CD4 + T-cell count of 467 cells/µliter, with a range of 366 to 1164 cells/µL. However, Patient 6, who had a low CD4 + T-cell count of 137 cells/µL, did not experience any more severe symptomatology compared to his peers, but did endure a prolonged duration of symptoms. Additionally, Patient 8 was diagnosed with pulmonary tuberculosis (PTB), and Patient 7 had a Hepatitis C Virus (HCV) infection. All patients with HIV were on antiretroviral therapy (ART), with either the bictegravir-tenofovir alafenamide-emtricitabine (BIC/FTC/TAF) combination regime or the two-drug regimen of dolutegravir (DTG) and lamivudine (3TC).

### Characterization of MPXV in the samples

Throughout the individual hospital stays of four of our patients, a total of 120 specimens were collected for analysis (Table 2). MPXV was detected most frequently in samples taken from the skin, where 59.3% of samples (32 out of 54 samples) tested positive. This was followed by oropharyngeal samples [50.0% (6 out of 12)], anal samples at 44.4% (4 out of 9), fecal samples at 18.1% (2 out of 11), and nasopharyngeal samples at 17.6% (3 out of 17). MPXV was not detected in blood samples (0 out of 5), urine samples (0 out of 11), or in semen (0 out of 1), as summarized in Table 2. This indicates that while the virus is present in skin, nasal, oral, and anal secretions, it is not typically found in blood or urine. Additionally, environmental testing (swabs from door handles, walls, floors, etc.) involved the collection of 925 samples, with MPXV being detected in 213 of these, resulting in a positive swab rate of 23.0%, suggesting the significant presence of MPXV virions in the surroundings.

**Table 2:** Detection of MPXV among the specimens of studied Mpox patients by qPCR

Patient	Pat. 01		Pat. 02		Pat. 03		Pat. 04			
Specimen site	All samples	Positive	All samples	Positive	All samples	Positive	All samples	Positive		Percentage of positive
Skin	38	21	3	1	4	3	9	7		59.3% (32/54)
Oropharyngeal	10	5	2	1	0	NT	0	NT		50.0% (6/12)
Anus	3	1	3	1	1	1	2	1		44.4% (4/ 9)
Feces	11	2	0	NT	0	NT	0	NT		18.1% (2/11)
Nasopharyngeal	10	1	2	0	3	2	2	0		17.6% (3/17)
Urine	11	0	0	NT	0	NT	0	NT		0 (0/11)
Blood	5	0	0	NT	0	NT	0	NT		0 (0 /5)
Seminal fluid	1	0	0	NT	0	NT	0	NT		0 (0/1)
Surroundings	868	210	34	3	23	0	8	1		23.0% (213/925)

NT: not tested. Surroundings: refers to areas in the patient’s room that were touched frequently, such as door handles, walls, floors, etc.

**Fig. 3:**
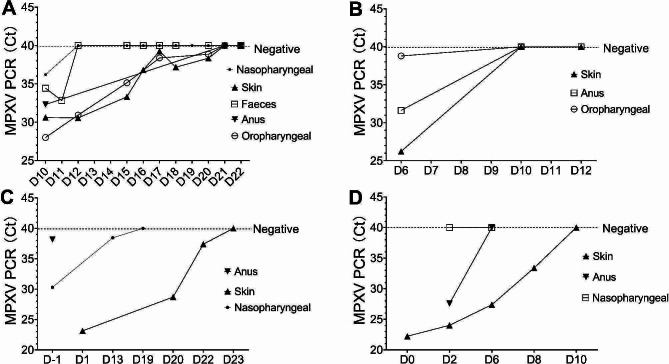
Viral loads and duration of MPXV detection among specimens in four Mpox patients All samples which were paired (i.e., taken from the same site in the same individual) were included in the analysis and were collected from Pat.01(**A**), Pat.02 (**B**), Pat.03 (**C**) and Pat.04 (**D**), respectively

With respect to the identification of MPXV, viral loads [which are inversely related to cycle threshold values] were assessed. Upon initial admission testing for MPXV, viral loads were highest in skin samples (median Ct of 26), followed by pharyngeal samples (median Ct of 32), and anal samples (median Ct of 33), as depicted in Fig. 3A-D. From the onset of symptoms, the median duration of detectable MPXV presence was 16 days in the skin samples (ranging from 10 to 23 days), 15.5 days in oral samples (ranging from 10 to 21 days), 11 days in nasal samples (ranging from 2 to 19 days), and 13 days in anal samples (ranging from 6 to 21 days). In Patient 1, MPXV was also identified in the fecal matter and persisted to day 11; however, eight consecutive tests for MPXV from day 11 onwards were negative (Fig. 3A). During the clinical treatment process, the viral loads of patients 5 through 9 were measured only once (specific Ct values can be seen in Table 3), therefore, we are unable to determine their trend of change.

**Table 3:** Viral loads of MPXV detection among specimens in Patient 5 to 9

Patient	Pat. 05		Pat. 06			Pat. 07			Pat. 08			Pat. 09			
Specimen site	Day	Ct		Day	Ct		Day	Ct		Day	Ct		Day	Ct	
Skin	D9	23		D6	26.48		D0	31.47		D5	27.07		D3	17.66	
Nasopharyngeal	D0	NT		D0	NT		D0	NT		D5	24.8		D3	24.75	

NT: not tested

## Discussion

In our retrospective examination of Biobank data, completed by October 11, 2023, we collated the data of nine individuals with PCR-confirmed MPXV infection. Our patient cohort presented with symptoms prompting them to seek healthcare, thus indicating the potential omission from our study of asymptomatic individuals or those with milder or minimal symptoms, who chose not to seek healthcare assistance. Our findings indicate that severe Mpox is associated with both a high burden of rash and a confluent rash distribution, corroborating previous research linking the extent of skin lesions to fever severity, symptom intensity, and illness duration [22]. The disease in our study group was typically mild to moderate in severity, with no recorded fatalities. Hospital stays generally ranged from two to three weeks.

The JYNNEOS vaccine, currently authorized for Mpox prevention during the ongoing outbreak in the US, has shown significant efficacy [23]. However, its availability remains limited in China. Research by Li et al. indicates that a majority of the Chinese population retains VTT-specific IgG antibodies for over 42 years following smallpox vaccination, potentially offering some protection against MPXV [24]. Nonetheless, all participants in our study were under 42 years old and reported no history of smallpox vaccination. Hence, further investigation into the efficacy of the vaccine against mpox is warranted in the future.

The cases studied occurred almost entirely among men who have sex with men, with seven out of nine patients being HIV-positive. Prior research indicates no significant differences in MPXV clinical manifestations between HIV-positive and HIV-negative MSM [17]. However, advanced HIV infection has been identified in other studies as a variable that is independently associated with more severe Mpox [22]. In our analysis, clinical presentation and immune status of afflicted patients appeared comparable between HIV-negative individuals and those living with HIV. The role of antiretroviral drugs, particularly nucleoside analogs like 3TC, FTC, and TAF, which inhibit DNA synthesis and terminate the proviral DNA chain, may contribute to the mitigation of lesion development. Cidofovir is a nucleotide analog of cytosine with potent activity against many DNA viruses. Cidofovir has already been used in animal experiments and in humans to treat a number of virally induced cutaneous infections including cowpox, camelpox, monkeypox, and molluscum contagiosum [25, 26]. However, the clinical application of cidofovir is frequently limited by nephrotoxity. Given that MPXV is a double stranded DNA virus, and it is likely that some ART drugs/nucleoside analogs may well inhibit MPXV replication. There is a possibility that these antiviral drugs may target MPXV DNA synthesis in a similar manner. Nonetheless, this requires further investigation in large clinical studies. Owing to the limited size of our study sample, additional controlled trial-based investigation is warranted to explore the relationship between Mpox and its clinical manifestations in a HIV co-infection context.

According to WHO guidelines, recommended specimens for testing can include materials from skin lesions such as swabs of lesional exudate and scabs, oropharyngeal swabs, rectal and/or genital swabs, as well as urine, semen, whole blood, serum, or plasma [27]. In our study, Mpox diagnosis was predominantly confirmed through swab samples obtained from skin and genital lesions, as well as from the throat or nasopharyngeal region, with blood testing being less frequently done. Our data indicates a higher prevalence of genital and anal lesions among patients. Furthermore, we observed variations in MPXV viral loads based on the collection site and the type of body fluid. Consistent with other investigations, the highest viral concentrations were found in skin lesion sites and oropharyngeal swabs [13, 28, 29].

For individuals with available follow-up PCR data, the longest duration for a lesion to continually test positive was 23 days post the onset of symptoms, suggesting a slower process of viral clearance. Based on this, oropharyngeal swabs and specimens from skin lesions are highly recommended for the laboratory confirmation of cases [30]. In our study, semen and serum were only tested in a single individual and yielded PCR-negative results, which deviates from observations obtained in other research [28, 31]. This discrepancy is most likely due to the fact that these tests were only done in one patient in our cohort. Moreover, MPXV was detected in nearly all reported cases within the oropharyngeal and anal regions [13, 29]. The presence of the virus in throat swabs suggests potential transmission through oral contact, including kissing and the exchange of saliva. Similarly, detection of the virus in anal and perianal lesions points to possible transmission via insertive anal intercourse. Higher viral loads in skin and mucosal sites, particularly in genital and anal areas, indicate that transmission is most likely during the close physical contact of sexual activity, and this may facilitate rapid spread between sexual partners. This observation concurs with those of recent studies that have reported high detection rates of MPXV in oropharyngeal and anal specimens, thus highlighting MPXV transmission pathways through oral and anal sexual practices.

To summarize, we evaluated the viral load at the onset of symptoms and monitored changes over time across various specimen types. Our analysis observed significantly higher viral loads in skin samples compared to throat, nasopharyngeal, blood, and semen samples. Although we observed a delay in viral clearance, this was largely influenced by the initial viral load detected. Further studies are warranted to ascertain whether certain ART drugs contribute to a decrease in lesion prevalence and promote beneficial outcomes in those with Mpox. Several limitations of our study need to be highlighted. Our case series is an observational series with infections confirmed by a PCR platform at our hospital. Our research cohort consists of only nine patients, of whom only two were HIV uninfected; this does not reflect the actual proportion of monkeypox-infected individuals in the general population. Individuals in this case series sought medical care because they were symptomatic or had HIV, meaning that asymptomatic, less symptomatic, or non-HIV-infected individuals may have been missed. Although our cohort, in which all nine patients were men who have sex with men, shows that the epidemic disproportionately affects men who have sex with men, it is important to emphasize that monkeypox is not a “homosexual disease”, and can affect anyone. Because all patients started noticing symptoms after symptom onset, early symptoms may have been underestimated. We urge physicians to be vigilant when examining any person presenting with an unusual acute rash, to avoid missing diagnoses of MPXV infection.

## Conclusion

This study enriches our comprehension of the transmission dynamics of MPXV among sexual partners. The elevated viral loads detected in skin and in mucosal areas, notably at genital and anal sites, imply that direct physical contact is the primary mode for transmission, rather than respiratory pathways or exposure to various body fluids. These insights may help in the development of preventive strategies and public health messaging around Mpox, and aid in tailoring and targeting these strategies to effectively reach and protect those most at risk of exposure to MPXV.

## Data Availability

The original contributions presented in the study are included in the article materials, further inquiries can be directed to the corresponding authors.
